# Beyond body mass index: phenotypic and circulating biomarkers for the identification and characterization of sarcopenic obesity

**DOI:** 10.3389/fnut.2026.1930821

**Published:** 2026-09-23

**Authors:** Maha Al Turki

**Affiliations:** 1 Department of Clinical Nutrition, College of Applied Medical Sciences, King Saud bin Abdulaziz University for Health Sciences, Riyadh, Saudi Arabia; 2 King Abdullah International Medical Research Centre, National Guard Health Affairs, Riyadh, Saudi Arabia

**Keywords:** anabolic resistance, biomarkers, body composition, muscle quality, myokines, myosteatosis, precision nutrition, sarcopenic obesity

## Abstract

Sarcopenic obesity (SO), the coexistence of excess adiposity with low skeletal muscle mass and function, is a growing but frequently underdiagnosed condition associated with physical disability, cardiometabolic disease, and increased mortality. Its recognition is hampered by an over-reliance on body mass index, which conceals muscle depletion, and by consensus diagnostic pathways that identify SO only once measurable functional impairment has developed. Advances in body-composition imaging and molecular biology may enable detection of subclinical abnormalities not captured by conventional anthropometric or functional assessment. This narrative review examines the phenotypic and circulating biomarkers proposed for the identification and characterization of SO. It first considers phenotypic markers of muscle quantity and, increasingly, muscle quality including myosteatosis quantified by computed tomography and the bioelectrical phase angle. It then surveys the molecular landscape: myokines and adipokines (myostatin, irisin, the leptin-to-adiponectin ratio), inflammatory mediators (such as tumor necrosis factor-α and interleukin-6) that drive anabolic resistance, and emerging metabolomic, lipidomic, and microRNA signatures. Drawing these together, we propose an integrated phenotype–biomarker framework for precision risk characterization, and consider its relevance in the era of highly effective pharmacological weight loss, where muscle preservation has become an urgent priority. Although most molecular markers remain investigational and lack standardized cut-offs, their integration with phenotypic assessment offers a promising route toward earlier, individualized identification of SO; assay standardization and prospective validation are the principal priorities for translation. The proposed framework is conceptual and has not been prospectively validated.

## 1 Introduction

The convergence of two global trends, population aging and the rising prevalence of obesity, has produced a clinically important but frequently overlooked phenotype in which excess adiposity coexists with progressive skeletal muscle depletion. This condition, termed sarcopenic obesity (SO), is not simply the sum of its parts: obesity and sarcopenia interact synergistically, with adipose-derived inflammation and lipotoxicity accelerating muscle catabolism while declining muscle mass and quality further impair metabolic regulation and physical function (1, 2). Individuals with SO carry a greater burden of cardiometabolic disease, physical disability, hospitalization, cognitive decline, and mortality than those with either obesity or sarcopenia alone (2, 3).

Despite growing recognition, the true burden of SO remains difficult to quantify, largely because prevalence estimates depend heavily on the diagnostic criteria applied. Pooled analyses report a global prevalence of roughly 11% in older adults, rising markedly after the age of 70, yet estimates in non-hospitalized older populations reach 14%, and analyses employing stricter definitions report figures as low as 7% (4, 5). These estimates are not directly comparable, as they derive from different populations, age ranges, and diagnostic definitions; the variation reflects methodological heterogeneity as much as true differences in burden. It nonetheless implies that a substantial proportion of affected individuals are never identified.

A central driver of this diagnostic gap is the enduring reliance on body mass index (BMI) as the primary indicator of nutritional and metabolic status. BMI cannot distinguish adipose from lean tissue, and in the context of obesity it actively conceals muscle depletion, so that clinically meaningful losses of muscle mass and quality remain invisible within a “normal” or elevated weight classification (2). The limitation is compounded by the recognition that individuals with identical anthropometric profiles may differ profoundly in visceral adiposity, ectopic fat deposition, and muscle composition. Of particular importance is myosteatosis, pathological lipid infiltration of skeletal muscle, which is increasingly implicated in impaired mobility, heightened morbidity, and poor survival, and is associated with adiposity, metabolic derangement, and mortality largely independent of muscle quantity (6). Muscle quality has thus emerged as a determinant of outcome at least as powerful as muscle mass itself.

Efforts to standardize diagnosis culminated in the 2022 joint consensus of the European Society for Clinical Nutrition and Metabolism and the European Association for the Study of Obesity (ESPEN–EASO), which established a two-stage framework combining obesity assessment with indicators of altered skeletal muscle function and body composition (7). This consensus represents a major advance. However, operationalization studies show that its two-step workflow, which positions functional impairment as the gateway to body-composition confirmation, is highly specific but of variable sensitivity, and that prevalence estimates differ substantially depending on which indices are applied (8). Because skeletal muscle possesses considerable neuromuscular compensatory capacity, microstructural and metabolic deterioration may be present before measurable declines in strength become apparent; requiring demonstrable functional impairment as a prerequisite may therefore overlook individuals with potentially relevant subclinical abnormalities.

This limitation has acquired new urgency in the era of highly effective pharmacological weight management. The rapid adoption of glucagon-like peptide-1 receptor agonists and dual agonists has transformed obesity treatment, but body-composition analyses indicate that a substantial fraction of the weight lost is lean soft tissue, making sensitive, early detection of muscle loss a contemporary clinical priority (discussed in Section 5.1).

In parallel, advances in metabolomics, lipidomics, and systems medicine have opened the possibility of detecting the biological processes underlying SO before they manifest as overt functional decline, focusing attention on the bidirectional endocrine crosstalk between skeletal muscle and adipose tissue mediated by myokines, adipokines, inflammatory cytokines, metabolite and lipid signatures, and circulating muscle-specific microRNAs (9). Although most remain investigational and lack standardized cut-offs, they offer a biologically informed complement to the structural and functional measures on which diagnosis currently rests.

Several recent reviews address overlapping ground. A 2025 systematic review and meta-analysis pooled biomarker effect sizes and distinguished shared from differential markers across sarcopenia and SO (10), and a 2025 narrative review examined digital technologies, wearables, and multi-omic data for scalable, remote precision diagnosis (11). The present review is distinct in being clinically and nutritionally oriented rather than technological or purely quantitative; in foregrounding the translational barriers that have impeded biomarker adoption and the muscle-preservation imperative of the GLP-1 era; and in explicitly separating SO-specific evidence from findings extrapolated from sarcopenia or obesity (Table 1). Rather than an operational, validated algorithm, we offer a conceptual scaffold intended to structure hypothesis-driven research and to identify individuals at risk of developing SO.

**Table 1:** Scope and contribution of recent reviews compared with the present article.

Review (year)	Type / focus	Biomarker treatment	Distinctive emphasis	SO-specific vs extrapolated
Feng et al. (10)	Systematic review & meta-analysis	Pooled effect sizes; shared vs. differential markers	Quantitative synthesis; inflammatory/hematologic	Distinguishes sarcopenia vs. SO statistically
Panagiotou and Brage (11)	Narrative review	Multi-omics as one component	Digital tools, wearables, eHealth, remote diagnosis	Not a focus
Present review (2026)	Narrative review + conceptual framework	Integrates phenotypic muscle-quality with circulating markers	Clinical nutrition; translational barriers; GLP-1 era	Explicitly separated (Tables 3, 4)

Accordingly, this review synthesizes current evidence on the phenotypic and circulating biomarkers investigated for the identification and characterization of SO in adults, characterizes the biological domains they represent, and proposes an integrated framework intended to support earlier, precision-based recognition of the condition.

### 1.1 Approach to the literature

This is a narrative review. Relevant literature was identified through searches of PubMed/MEDLINE, Scopus, and Web of Science from database inception through July 2026, using combinations of the terms ‘sarcopenic obesity,’ ‘sarcopenia,’ ‘obesity,’ ‘body composition,’ ‘myosteatosis,’ ‘phase angle,’ ‘biomarker,’ ‘myokine,’ ‘adipokine,’ ‘metabolomics,’ ‘lipidomics,’ and ‘microRNA,’ supplemented by hand-searching of reference lists. Only English-language studies in adults were considered. Priority was given to studies in adults with SO (study-defined or consensus-defined); where SO-specific data were unavailable, evidence extrapolated from sarcopenia or obesity is identified as such. Source selection was guided by relevance and domain expertise rather than a registered protocol, and the review does not claim the completeness of a systematic or scoping review — a limitation stated in Section 5.4.

## 2 Phenotypic assessment: from muscle quantity to muscle quality

### 2.1 The ESPEN–EASO framework and its structural constraints

The 2022 ESPEN–EASO consensus established a standardized two-stage pathway in which screening (elevated BMI or waist circumference plus sarcopenia risk factors) is followed by assessment of altered skeletal muscle function and then confirmatory body-composition measurement (7). From the standpoint of early detection, the sequence embeds a structural constraint: because demonstrable functional impairment gates entry to body-composition confirmation, individuals whose muscle has begun to deteriorate qualitatively but who retain compensated strength may not proceed to assessment (Figure 1). Throughout this review we distinguish four related but non-equivalent states: (i) consensus-diagnosed SO (impaired muscle function plus altered body composition per ESPEN–EASO); (ii) obesity with impaired muscle quality but preserved function, which does not meet current diagnostic criteria; (iii) a preclinical or ‘at-risk’ phenotype defined by early molecular or imaging abnormalities; and (iv) prediction of future SO. The proposition that molecular or muscle-quality abnormalities precede functional decline is biologically plausible but, absent longitudinal data linking these markers to subsequent loss of strength, physical performance, or clinical outcomes, remains a hypothesis rather than an established diagnostic principle. The framework proposed here therefore targets identification of individuals at risk of developing SO, complementing rather than replacing the consensus criteria.

**Figure 1:**
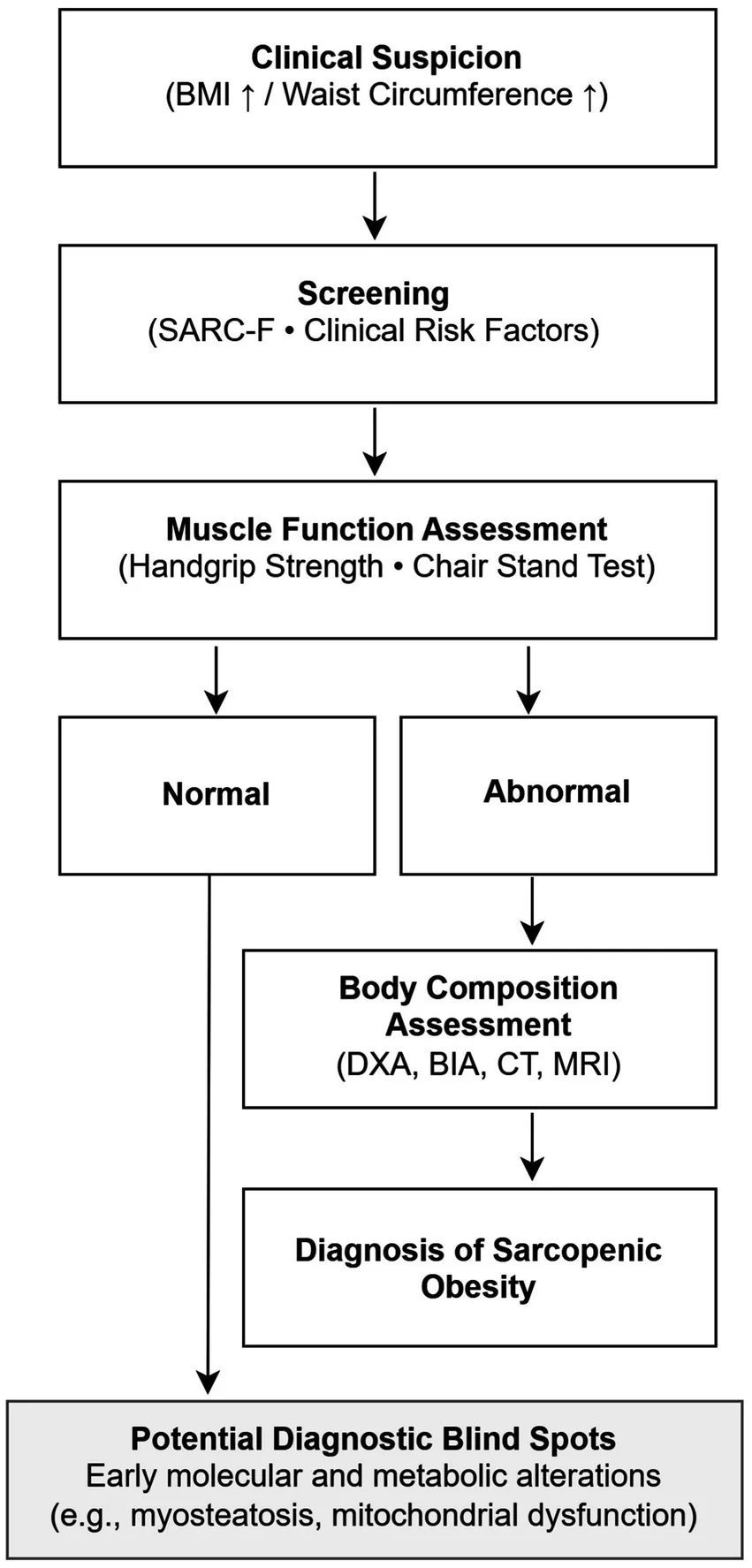
The current ESPEN–EASO diagnostic pathway for sarcopenic obesity and its principal blind spots. Clinical suspicion (elevated BMI or waist circumference) prompts screening (e.g., SARC-F and clinical risk factors) and muscle-function assessment; only abnormal results proceed to body-composition assessment and confirmatory diagnosis. Because functional impairment gates entry to body-composition confirmation, early molecular and metabolic alterations, including myosteatosis and mitochondrial dysfunction, may remain undetected. BIA, bioelectrical impedance analysis; BMI, body mass index; CT, computed tomography; DXA, dual-energy X-ray absorptiometry; MRI, magnetic resonance imaging; SARC-F, Strength, Assistance with walking, Rising from a chair, Climbing stairs and Falls questionnaire.

### 2.2 Body-composition modalities and their trade-offs

The modalities used to resolve tissue compartments differ substantially in what they measure, their accessibility, and their cost, and no single technique is optimal across all settings (Table 2). Dual-energy X-ray absorptiometry (DXA) is widely available and low in radiation and is embedded in current criteria, but it indexes tissue quantity and cannot reliably distinguish contractile muscle from intramuscular fat, with accuracy further degraded in severe obesity and altered hydration (2). Bioelectrical impedance analysis (BIA) is inexpensive and portable but sensitive to hydration, inflammation, and fluid redistribution. Computed tomography (CT) and magnetic resonance imaging (MRI) provide the most complete characterization of muscle morphology and adipose distribution and quantify muscle radiation attenuation, an established surrogate of muscle quality; attenuation is not a direct measurement of intramyocellular lipid and is influenced by technical factors including tube voltage, contrast phase, reconstruction algorithm, and tissue edema, which complicate cross-study comparison. CT and MRI are consequently regarded as reference methods for muscle morphology and composition, although DXA-derived appendicular lean mass remains the mass index embedded in current consensus algorithms; their principal constraints are cost, access, and, for CT, radiation (12).

**Table 2:** Comparative evaluation of body-composition modalities for the assessment of muscle quantity and quality.

Modality	Primary measurements	Muscle quality / myosteatosis	Advantages	Key limitations
DXA	Appendicular lean mass, regional fat, bone mineral density	Low: indexes quantity; cannot separate muscle from intramuscular fat	Widely available, low radiation; embedded in consensus criteria	Areal data; cannot separate visceral from subcutaneous fat; degraded in severe obesity/altered hydration
BIA	Fat-free mass, fat mass, total body water, phase angle	Moderate: phase angle proxies cell-membrane integrity	Inexpensive, portable; bedside screening	Sensitive to hydration, inflammation; device/equation dependent
CT	Muscle cross-sectional area, radiation attenuation (HU), visceral/intermuscular fat	High: attenuation is an established surrogate of myosteatosis	High accuracy; resolves fat distribution; amenable to automated analysis	Ionizing radiation; cost/access; attenuation thresholds not standardized; technical confounders
MRI	Muscle volume, intramuscular fat fraction, adipose distribution	Very high: quantitative fat-fraction mapping, no radiation	Reference standard for composition; no radiation	High cost, limited access, longer acquisition

BIA, bioelectrical impedance analysis; CT, computed tomography; DXA, dual-energy X-ray absorptiometry; HU, Hounsfield units; MRI, magnetic resonance imaging.

A notable contemporary development is the move toward opportunistic and automated body-composition analysis: deep-learning segmentation of routinely acquired CT (typically at the third lumbar vertebra) now enables extraction of skeletal muscle area, muscle radiation attenuation, and visceral-to-subcutaneous fat ratios without additional scanning or cost, with comparable population-scale phenotyping emerging for MRI. These approaches may substantially lower the practical barrier to muscle-quality assessment.

### 2.3 Muscle quality as the emerging phenotypic priority

Throughout this review, muscle quality is used as an umbrella term for non-quantitative characteristics of skeletal muscle that influence its functional and metabolic performance. These include tissue composition, particularly intramuscular and intermuscular fat infiltration (myosteatosis), assessed using CT attenuation or MRI-derived fat measures; and cellular integrity, for which bioelectrical phase angle provides an indirect, hydration-sensitive marker. Metabolic characteristics such as mitochondrial function represent an additional biological dimension but are not directly measured by either CT attenuation or phase angle. Accordingly, these measures capture complementary rather than interchangeable aspects of muscle quality, and the specific facet assessed is stated throughout the review.

A consistent theme across recent evidence is that muscle quality — composition, lipid infiltration, and metabolic capacity — predicts clinical outcomes at least as strongly as muscle mass, such that individuals with identical mass indices may differ markedly in function and survival according to underlying tissue integrity (2). Two markers are especially relevant to bridging the diagnostic gap. Myosteatosis, quantified on CT as reduced skeletal muscle radiation attenuation, is associated with insulin resistance, systemic inflammation, and increased mortality largely independent of total muscle mass or BMI (6, 13); it has been proposed as a candidate indicator of early tissue deterioration, though prospective confirmation that it precedes functional decline in SO is lacking, and diagnostic thresholds remain non-standardized across studies. The bioelectrical phase angle (PhA), derived from multifrequency BIA, reflects cell-membrane integrity and body cell mass, with lower values indicating compromised cellular health (14), and shows good accuracy for detecting sarcopenia and predicts low muscle strength, impaired quality of life, and mortality across clinical populations (15). PhA is, however, influenced by the measurement device and frequency, hydration status, age, sex, BMI, and ethnicity; these are particularly relevant in obesity, where altered hydration and adiposity may confound interpretation, so population- and device-specific reference values are required. Because it captures cellular-level change, PhA has been proposed as an early indicator, though prospective evidence that it precedes mass or functional loss in SO specifically remains limited.

## 3 The molecular biomarker landscape

Because molecular abnormalities may accompany or potentially precede structural and functional manifestations of SO, circulating biomarkers offer a window onto disease biology that conventional imaging and function tests may not fully capture. Importantly, for most markers the direct evidence in consensus-defined SO is limited, cross-sectional, and derived from small, heterogeneously defined cohorts; much of the supporting data are extrapolated from sarcopenia or obesity, and are identified as such in Tables 3, 4 and in the text.

**Table 3:** Myokine, adipokine, and inflammatory biomarkers implicated in sarcopenic obesity, with SO-specific evidence.

Biomarker	Direction	Evidence context	SO-specific evidence (study; definition; *n*; replication)	Interpretation	Ref.
Myostatin	↓ early SO; ↑ obesity/IR	SO-specific (single) + extrapolated	Ishibashi et al. (16): 432 women 30–59 y; study-defined (SMI < 5.7 + fat≥30%); SO *n* = 27; ↓ serum. Single, sex-restricted, unreplicated.	Direction unresolved	(16)
Irisin	↓	SO-specific + sarcopenia	Kim et al. (18) (Hallym cohort; cross-sectional+longitudinal) and Oguz et al. (19) (90 T2DM; FM/FFM criteria): both ↓.	Directionally consistent across limited SO-specific studies; external replication limited	(18, 19)
Leptin	↑	Extrapolated (obesity)	No convincing SO-specific validation.	Adiposity-driven	(1)
Adiponectin	↓ / inconsistent*	SO-specific + sarcopenia	Kim et al. (18) (Hallym): paradoxical/inconsistent associations.	Interpret vs. adiposity	(18)
Leptin-to-adiponectin ratio	↑ (hypothesized)	Extrapolated (obesity)	No SO-specific validation.	Composite; unvalidated in SO	(18)
TNF-α	↑	Sarcopenia/SO-associated	Pooled in Feng et al. (10) meta-analysis (sarcopenia/SO).	Non-specific mediator	(10, 21)
IL-6	↑ (chronic)	Sarcopenia/SO-associated	Pooled in Feng et al. (10); IL-6–sarcopenia meta-analyses.	Context-dependent	(10, 21)
CRP	↑	Extrapolated (sarcopenia/frailty)	Limited SO-specific data.	Accessible; non-specific	(10, 35)

“SO-specific” denotes studies in SO cohorts, whether study-defined or consensus (ESPEN–EASO)-defined, as indicated. *Adiponectin shows inconsistent (‘paradoxical’) associations. Where SO-specific evidence is limited or absent, the reported direction is extrapolated or hypothetical rather than demonstrated in SO. CRP, C-reactive protein; IR, insulin resistance; SO, sarcopenic obesity; TNF-α, tumor necrosis factor-α.

**Table 4:** Multi-omic (metabolomic, lipidomic, and transcriptomic) signatures explored in sarcopenic obesity, with evidence context.

Domain	Marker(s)	Pattern	Evidence context	SO-specific evidence	Ref.
Metabolomic	BCAAs	↑ obesity/IR; ↓ some sarcopenia	Extrapolated; SO direction unsettled	No SO-specific validation	(10, 24)
Metabolomic	3-Methylhistidine	↑ (hypothesized)	Mechanistic/sarcopenia	No SO-specific validation	(35)
Lipidomic	Ceramides	↑ (extrapolated)	Mechanistic/obesity-IR	No SO-specific validation	(26)
Lipidomic	Acylcarnitines (C3/C5)	↑ (extrapolated)	Mechanistic/obesity-IR	No SO-specific validation	(24)
Transcriptomic	Myo-miRs (miR-1,-133a/b,-206,-486)	Mixed (miR-486-5p ↑)	SO pilot + sarcopenia	Pilot in SO (small, exploratory, unreplicated); Dowling et al. (28) review	(27, 28)

BCAA, branched-chain amino acid; IR, insulin resistance; miR, microRNA; SO, sarcopenic obesity.

### 3.1 Myokines and adipokines

Skeletal muscle and adipose tissue communicate through secreted myokines and adipokines, and shifts in their profiles are among the earlier reported changes in SO and related conditions, although the temporal sequence relative to functional decline has not been firmly established. Myostatin, a negative regulator of muscle mass that suppresses Akt/mTOR signaling and promotes ubiquitin–proteasome-mediated proteolysis, illustrates the complexity of biomarker translation: its circulating directionality is inconsistent, with elevation reported in obesity and insulin resistance but reduction reported in early-stage SO (16) — plausibly reflecting differences in disease stage and assay platform. The apparently divergent findings for circulating myostatin probably reflect both biological and analytical heterogeneity. Circulating concentrations may be influenced by total muscle mass, adiposity and insulin resistance, age and sex, and regulation of myostatin production and bioavailability. Analytical factors further complicate comparison because assays differ in their recognition of mature and latent forms, and some immunoassays show cross-reactivity with structurally related TGF-β-family ligands such as GDF11 (17). Thus, lower concentrations reported in some SO cohorts should not necessarily be interpreted as contradicting obesity-associated increases; rather, the net circulating signal may vary according to phenotype, disease context, and measurement method. Longitudinal studies using standardized assays are required to determine whether a reproducible stage-dependent pattern exists. Irisin, cleaved from FNDC5 with muscle contraction, promotes adipose browning, mitochondrial biogenesis, and insulin sensitivity; reduced circulating irisin is reported in SO across limited cross-sectional studies (18–20). Dysfunctional adipose tissue overproduces leptin while suppressing the insulin-sensitizing adipokine adiponectin, and the leptin-to-adiponectin ratio is proposed as a composite index of adipocyte dysfunction, although adiponectin itself shows inconsistent, sometimes paradoxical associations with sarcopenia (the ‘adiponectin paradox’) (18). These markers, and the SO-specific evidence supporting them, are summarized in Table 3.

### 3.2 Inflammatory biomarkers and immunometabolic dysfunction

Chronic low-grade inflammation is widely regarded as a central mechanistic link between excess adiposity and muscle degradation, establishing SO as an immunometabolic disorder (21). Hypertrophied visceral adipocytes recruit and polarize M1 macrophages, sustaining release of TNF-α and IL-6, which activate the nuclear factor-κB (NF-κB) pathway in skeletal muscle. Beyond driving proteolysis, this inflammatory milieu induces anabolic resistance through inhibitory serine phosphorylation of insulin receptor substrate-1 (IRS-1) at residues such as Ser307, suppressing Akt/mTOR signaling. The two principal muscle-specific E3 ubiquitin ligases are regulated by distinct routes: MuRF1 is induced by TNF-α largely via NF-κB, whereas Atrogin-1/MAFbx is controlled predominantly by FoxO transcription factors, which become active when Akt signaling is suppressed (20); inflammation and anabolic resistance therefore converge on atrogene induction through two complementary arms (Figure 2). One caveat merits emphasis: IL-6 is context-dependent, contraction-induced IL-6 being insulin-sensitizing whereas chronically elevated IL-6 is catabolic, and C-reactive protein, downstream of IL-6, offers an accessible if non-specific readout. Clinically, acute exercise-associated and chronic inflammatory IL-6 signals are distinguished primarily by sampling context and temporal pattern rather than by a validated concentration threshold. Exercise induces a transient rise in circulating IL-6, the magnitude of which depends on exercise duration and intensity, followed by a decline during recovery (23). In contrast, chronic metabolic inflammation is characterized by persistently elevated resting IL-6 in the absence of recent strenuous exercise or acute illness and may occur alongside elevation of other inflammatory markers such as C-reactive protein. Accordingly, interpretation of IL-6 as a candidate biomarker in SO requires standardized resting sampling, documentation of recent exercise and intercurrent inflammatory conditions, and, where clinically or analytically relevant, repeated measurements and assessment of the broader inflammatory profile. No validated IL-6 threshold currently distinguishes exercise-derived from chronic inflammatory signaling in SO.

**Figure 2:**
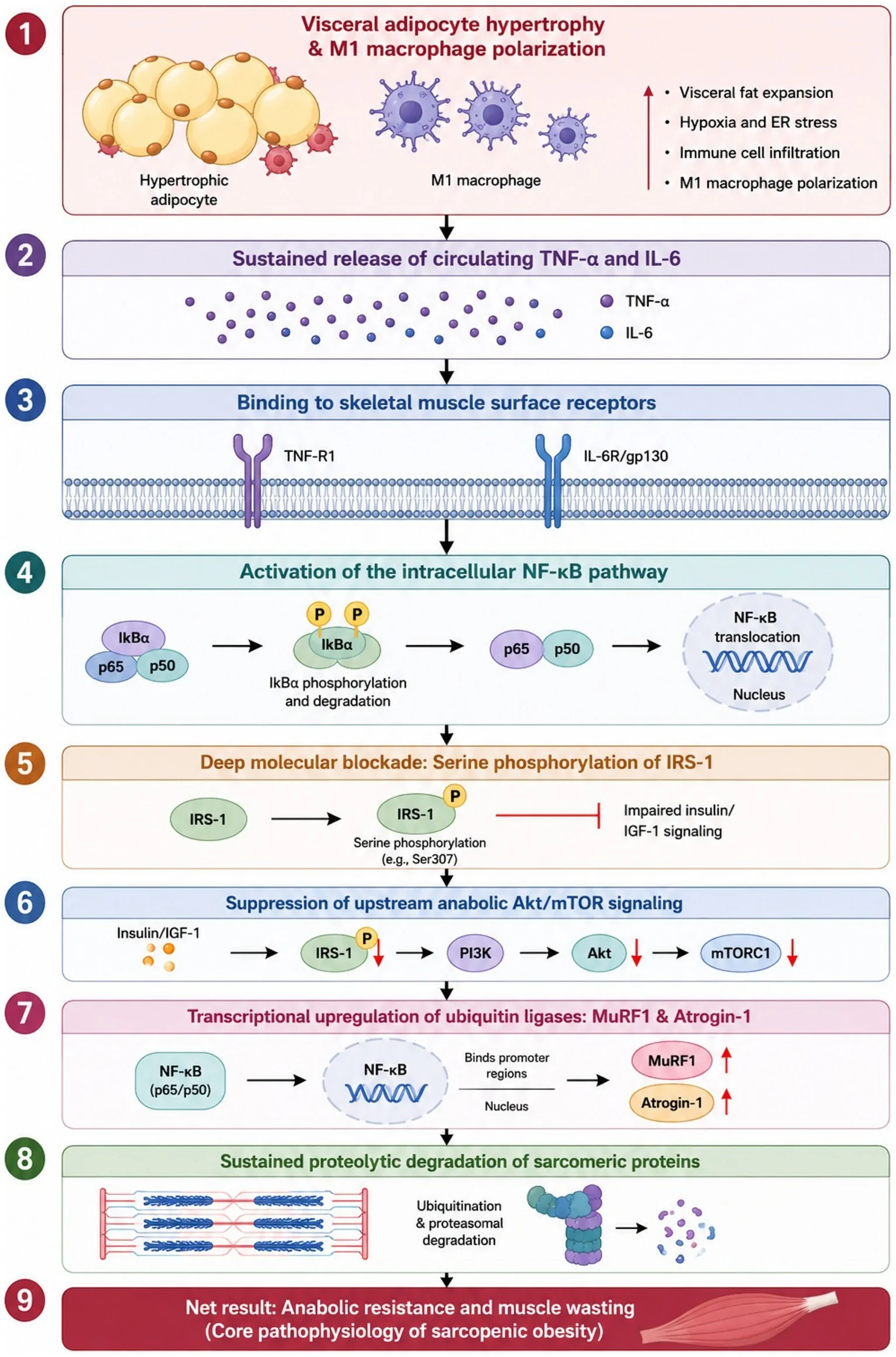
The immunometabolic cascade linking visceral adipose-tissue inflammation to skeletal-muscle proteolysis and anabolic resistance in sarcopenic obesity. Adipocyte hypertrophy and M1 macrophage polarization drive sustained TNF-α and IL-6 release, which bind muscle receptors and activate NF-κB, promote inhibitory serine phosphorylation of IRS-1 (Ser307), suppress PI3K–Akt–mTORC1 signaling, and up-regulate MuRF1 (principally NF-κB-dependent) and Atrogin-1/MAFbx (principally FoxO-dependent, following Akt suppression), driving ubiquitin–proteasome degradation of sarcomeric proteins and culminating in anabolic resistance and muscle wasting. For visual clarity, atrogene regulation is depicted in simplified form; MuRF1 is regulated principally by NF-κB and Atrogin-1/MAFbx principally by FoxO. ↑, increased; ↓, decreased; P, phosphorylation; ⊣, inhibition. Initial draft generated using OpenAI ChatGPT (GPT-5.5) and edited by the author.

### 3.3 Metabolomic and lipidomic signatures

High-throughput mass spectrometry has revealed metabolic signatures associated with the SO phenotype. Circulating branched-chain amino acids (BCAAs) are elevated in obesity and insulin resistance, yet chronically elevated levels mark, and may mechanistically contribute to, insulin resistance — attributed partly to excess oxidation generating branched-chain keto acids and short-chain (C3/C5) acylcarnitines that promote lipotoxicity (24). Their directionality in SO is not settled, however, as some sarcopenia cohorts report lower circulating BCAAs. The apparently divergent BCAA patterns may similarly reflect the competing biology of obesity and muscle depletion. In obesity and insulin resistance, elevated circulating BCAAs have been linked to impaired tissue-specific BCAA catabolism, including dysregulation of the branched-chain α-ketoacid dehydrogenase pathway in adipose tissue and liver (25). In sarcopenia, lower circulating concentrations reported in some cohorts may instead reflect reduced dietary protein intake, age-related changes in protein turnover, diminished muscle mass, or differences in metabolic utilization. Consequently, the BCAA profile in SO may represent the net effect of opposing adiposity-related and muscle-related influences rather than a uniform disease-specific signature. Acylcarnitines more broadly signal incomplete mitochondrial β-oxidation, while ceramides accumulate ectopically in muscle, disrupt IRS-1 signaling, and promote lipotoxic injury (26). 3-Methylhistidine, released on breakdown of actin and myosin, is a classical index of myofibrillar proteolysis, though confounded by dietary meat intake. These platforms are mapped in Table 4.

### 3.4 MicroRNA signatures

Circulating muscle-enriched microRNAs (myo-miRs), including miR-1, miR-133a/b, miR-206, and miR-486, regulate satellite-cell proliferation, myogenesis, and regeneration through the IGF-1/Akt/FoxO axis, and are stable in biofluids (27). As candidate SO biomarkers they remain exploratory: directionality is inconsistent and is modulated by exercise and protein intake. A pilot screening in patients meeting ESPEN–EASO criteria found increased circulating miR-486-5p in SO, whereas myo-miRs are reduced in other sarcopenia contexts; the microRNA evidence common to obesity, sarcopenia, and SO is reviewed systematically in reference 28, and these markers require phenotype-specific validation before clinical use (Table 4).

## 4 An integrated phenotype–biomarker framework

No single marker, phenotypic or molecular, is likely to capture the biological complexity of SO. The measures reviewed here are complementary: phenotypic tools quantify the structural and functional consequences of the disease, while circulating biomarkers report the underlying processes — inflammation, anabolic resistance, lipotoxicity, mitochondrial dysfunction, and tissue remodeling — that drive them and that may become detectable earlier. We therefore propose an integrated framework (Figure 3) in which comprehensive clinical evaluation combines phenotypic assessment (anthropometry, body composition, muscle-quality imaging, and functional testing) with a molecular panel, synthesized into an integrated biological and clinical characterization that distinguishes individuals at risk of developing SO from those with consensus-diagnosed SO and guides personalized, multidisciplinary management and longitudinal monitoring. We emphasize that Figure 3 is a conceptual scaffold rather than a validated risk-characterization instrument: the specific input variables, thresholds, weighting, outcome anchors, and the prospective validation required to establish predictive performance remain to be defined. The framework is intended to identify individuals at risk and to support monitoring, not as an alternative to the consensus diagnostic criteria.

**Figure 3:**
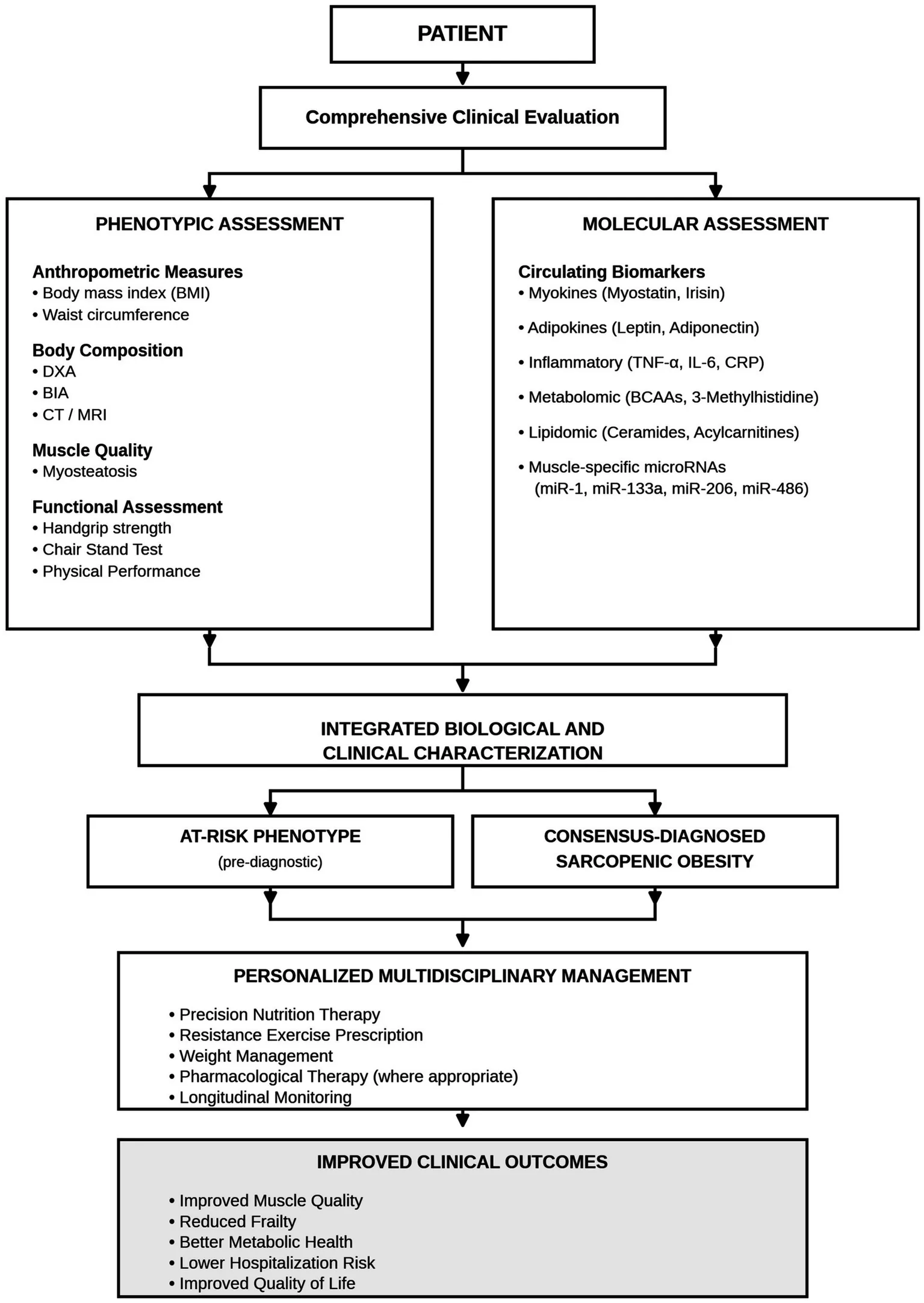
Conceptual framework integrating phenotypic and molecular biomarkers for the identification, characterization, and longitudinal monitoring of sarcopenic obesity. Comprehensive clinical evaluation combines phenotypic assessment (anthropometric, body-composition, muscle-quality, and functional measures) with molecular assessment; integration supports biological and clinical characterization, distinguishing individuals at risk of developing SO from those with consensus-diagnosed SO, and guides personalized management and longitudinal monitoring. The framework is conceptual and has not been prospectively validated. BCAA, branched-chain amino acid; BMI, body mass index; CRP, C-reactive protein; CT, computed tomography; DXA, dual-energy X-ray absorptiometry; IL-6, interleukin-6; miR, microRNA; MRI, magnetic resonance imaging; TNF-α, tumor necrosis factor-α.

To illustrate how multidimensional information might be integrated, an individual with obesity but preserved measured muscle function who shows concordant abnormalities across domains — for example, reduced CT muscle attenuation together with an elevated leptin-to-adiponectin ratio and persistently elevated resting inflammatory markers — could be considered a higher-concern, at-risk profile for research and closer clinical observation, whereas isolated or discordant abnormalities may carry less biological concern. This example is illustrative rather than prescriptive: the specific variables, thresholds, weighting, and outcome anchors have not been established, and their derivation and prospective validation are prerequisites before such an approach could be used for clinical risk classification.

## 5 Discussion and future directions

### 5.1 Muscle preservation in the era of pharmacological weight loss

Two developments give this integrative approach particular contemporary relevance. The first is the transformation of obesity care by glucagon-like peptide-1 receptor agonists and dual GLP-1/GIP agonists. Although these agents produce unprecedented weight loss, a substantial fraction of the weight lost is DXA-derived lean soft tissue. Lean soft tissue is not synonymous with skeletal muscle mass, and a reduction in lean mass does not by itself indicate sarcopenia or loss of muscle strength or function; whether these changes translate into functional decline requires direct assessment of strength and physical performance (29, 30). Some analyses suggest that the most potent agents may preserve lean mass least, although appropriately powered, head-to-head comparisons using muscle-specific measures are limited (29). The picture is nuanced rather than uniformly adverse: relative lean-mass percentage may be maintained, and in supervised settings combining pharmacotherapy with adequate protein and resistance exercise the prevalence of SO can fall as muscle function improves (30). Precisely because outcomes hinge on whether muscle is protected, sensitive markers of muscle quantity and quality are needed to identify patients at risk of iatrogenic SO and to monitor them over time.

### 5.2 Reframing management around oral medical nutrition therapy

These considerations also reframe management. First-line medical nutrition therapy for SO is oral rather than artificial: adequate energy with a controlled deficit, elevated protein intake individualized toward approximately 1.2–1.5 g/kg/day, leucine-enriched meal distribution, and repletion of vitamin D and long-chain omega-3 fatty acids, combined with resistance exercise to counter anabolic resistance (31–33). These targets derive largely from guidelines and trials in older adults and sarcopenia (PROT-AGE; ESPEN) rather than SO-specific intervention trials, and require individualization; protein targets in particular must be tempered in patients with chronic kidney disease or other contraindications, and the SO-specific evidence base remains limited. Enteral or parenteral support is reserved for patients unable to meet requirements orally. The role of branched-chain amino acid supplementation is biologically rational but unresolved, and interventional benefits on muscle outcomes have been inconsistent (24).

### 5.3 Why candidate biomarkers have not yet reached the clinic

Despite two decades of candidate biomarkers, none is yet embedded in routine clinical practice for SO. First, no single marker captures a syndrome spanning muscle mass, quality, strength, and metabolic function; expert bodies have long favored combined, multi-domain panels over any single analyte (34, 35). Second, analytical and pre-analytical barriers are substantial: circulating concentrations fluctuate with circadian rhythm, fasting status, and recent physical activity, and are sensitive to sample handling, while assay and inter-laboratory variability and the absence of standardized reference ranges impede comparison (35). Third, specificity is a persistent problem: C-reactive protein, interleukin-6, and tumor necrosis factor-α reflect generalized inflammation and aging rather than muscle-specific pathology, and are confounded by adiposity, renal and hepatic function, comorbidity, and medication; a recent umbrella review concluded that the diagnostic accuracy of current biomarkers does not yet meet the standard required for clinical implementation (36). Fourth, until the 2022 ESPEN–EASO consensus there was no harmonized definition of SO, so earlier biomarkers were assessed against inconsistent phenotypes (7). These challenges are compounded in SO specifically, because obesity perturbs many of the same molecules — leptin, C-reactive protein, adiponectin, and BCAAs track adiposity and insulin resistance as much as muscle status — so isolating a muscle-specific signal is harder than in sarcopenia alone (18, 24). Progress therefore requires validated multi-marker panels interpreted alongside phenotype, built on standardized assays and SO-specific reference ranges and anchored to prospective outcomes.

### 5.4 Limitations

Several limitations must be acknowledged. Most biomarker evidence is cross-sectional and associative rather than prospective; assays and cut-offs are not standardized, and reference values for markers such as CT muscle attenuation and phase angle remain unsettled; and directionality is inconsistent for several markers, most notably myostatin, the branched-chain amino acids, and the myo-miRs. Much of the available evidence derives from sarcopenia or obesity populations rather than the combined SO phenotype, so extrapolation must be cautious. This review is itself narrative rather than systematic: it was not conducted with a registered protocol or exhaustive search, and the proposed framework is conceptual and has not been prospectively validated.

### 5.5 Future directions

Priorities for translation follow directly. Assay harmonization and the establishment of SO-specific reference ranges are prerequisites for clinical use. Prospective, longitudinal, and ideally multi-omic studies are needed to determine which markers precede functional decline and predict outcomes, and to test whether biomarker-guided characterization improves them. Integration of molecular panels with automated, opportunistic imaging-derived muscle-quality metrics, and formal validation of the framework in prospective cohorts and intervention trials — including studies of muscle preservation during pharmacological weight loss — represent the most valuable next steps.

## 6 Conclusion

Sarcopenic obesity is a clinically consequential yet under-recognized condition whose detection is constrained by the limitations of body mass index and by diagnostic pathways that act only once functional impairment is established. Phenotypic markers of muscle quality, myosteatosis and the bioelectrical phase angle, together with a growing panel of circulating myokine, adipokine, inflammatory, metabolomic, lipidomic, and transcriptomic biomarkers, offer complementary means of illuminating subclinical biological processes that may not be captured by conventional measures of muscle quantity and function. Although these markers are not yet ready for routine use, their integration with phenotypic assessment within a precision-oriented framework offers a credible path toward earlier identification and individualized management of SO. Future assessment paradigms may increasingly integrate phenotypic and molecular profiling to identify individuals at heightened risk of developing sarcopenic obesity, characterize biological heterogeneity among those with established disease, and support longitudinal monitoring before substantial functional deterioration occurs.

## Glossary

ALM: appendicular lean mass
Akt: protein kinase B
BCAA: branched-chain amino acid
BCKA: branched-chain keto acid
BIA: bioelectrical impedance analysis
BMI: body mass index
CRP: C-reactive protein
CT: computed tomography
DXA: dual-energy X-ray absorptiometry
EASO: European Association for the Study of Obesity
ESPEN: European Society for Clinical Nutrition and Metabolism
FNDC5: fibronectin type III domain-containing protein 5
FoxO: forkhead box O
GIP: glucose-dependent insulinotropic polypeptide
GLP-1: glucagon-like peptide-1
HU: Hounsfield units
IGF-1: insulin-like growth factor-1
IL-6: interleukin-6
IRS-1: insulin receptor substrate-1
miR/miRNA: microRNA
MRI: magnetic resonance imaging
mTORC1: mechanistic target of rapamycin complex 1
MuRF1: muscle RING-finger protein-1
NF-κB: nuclear factor-κB
PhA: phase angle
PI3K: phosphoinositide 3-kinase
SARC-F: Strength, Assistance with walking, Rising from a chair, Climbing stairs and Falls
SMI: skeletal muscle index
SO: sarcopenic obesity
TNF-α: tumor necrosis factor-α
TNF-R1: TNF receptor 1
VAT/SAT: visceral-to-subcutaneous adipose tissue ratio
